# *In* *vitro* antifungal susceptibilities of *Candida* species to liposomal amphotericin B, determined using CLSI broth microdilution, and amphotericin B deoxycholate, measured using the Etest

**DOI:** 10.1099/jmm.0.000402

**Published:** 2017-02-24

**Authors:** Grazia Lovero, Osvalda De Giglio, Serafina Rutigliano, Giusy Diella, Giuseppina Caggiano, Maria Teresa Montagna

**Affiliations:** Department of Biomedical Science and Human Oncology, Hygiene Section, Università degli Studi of Bari ‘Aldo Moro’, Bari, Italy

**Keywords:** *Candida*, liposomal amphotericin B, CLSI broth microdilution, amphotericin B deoxycholate, E-test

## Abstract

The antifungal susceptibilities of 598 isolates of *Candida* spp. (bloodstream and other sterile sites) to liposomal amphotericin B (L-AmB) *versus* amphotericin B (AmB) were determined. MICs were calculated using the Clinical and Laboratory Standards Institute broth microdilution (M27-A3) method for L-AmB and the Etest method for AmB. The MIC_50_/MIC_90_ (µg ml^−1^) values for L-AmB broth microdilution and AmB Etest were 0.25/1 and 0.19/0.5, respectively. The overall essential agreement (±2 dilutions) was 91.5 %, ranging from 37.5 % (*Candida lusitaniae*) to 100 % (*Candida glabrata* and *Candida krusei*). Categorical agreement between the two methods was categorized based on a previously published breakpoint (susceptible/resistant MIC cut-off of 1 µg ml^−1^). The overall categorical agreement at the 48 h reading was 97.3 %, ranging from 72.7 % (*C. krusei*) to 100 % (*Candida albicans*). Major and very major discrepancies occurred in 2.3 and 0.3 %, respectively. Spearman’s ρ was 0.48 (*P*<0.0001). These results demonstrate the utility of the AmB Etest as a surrogate marker to predict the sensibility and resistance of *Candida* spp. to L-AmB and thus to support its use in antifungal treatment.

## Introduction

*Candida* spp. are important causative agents of invasive fungal infections. They are associated with significant morbidity, prolonged hospital stays, high mortality and increased healthcare costs. Among the currently available antifungals, amphotericin B (AmB), a polyene macrocyclic, has long been the drug of choice for many life-threatening invasive fungal infections. Its broad spectrum of activity and the virtual absence of resistance account for its continued importance [1]. However, the clinical use of AmB is impaired by its safety profile. Its associated adverse effects include acute kidney [2, 3], which is mediated by vasoconstriction and direct tubular toxicity. To attenuate the adverse effects of AmB, lipid-based formulations have been developed, including liposomal amphotericin B (L-AmB), amphotericin B lipid complex and amphotericin B colloidal dispersion. Of these, L-AmB appears to be substantially less toxic in terms of nephrotoxicity and the incidence of infusion-related adverse events [4, 5]. Based on its enhanced safety and efficacy profile, the use of L-AmB as empirical antifungal therapy in febrile neutropenic patients is recommended by the guidelines of the US Food and Drug Administration (www.fda.gov), the European Society of Clinical Microbiology and Infectious Diseases [6] and the European Conference on Infections in Leukaemia [7]. L-AmB is also recommended (strong recommendation; moderate-quality evidence) as therapy for neutropenic patients with candidaemia according to the guidelines of the Infectious Diseases Society of America [8].

Until now, *in vitro* data on the activity of L-AmB compared with that of AmB against clinical isolates of *Candida* spp. are lacking [9–11], despite a larger number of studies indicating that L-AmB is non-inferior to AmB in terms of *in vivo* efficacy [4, 5, 12, 13]. We recently performed a head-to-head challenge of L-AmB and AmB against 604 clinical yeast isolates using the Clinical and Laboratory Standards Institute (CLSI) broth microdilution (BMD) M27-A3 method, showing a high level of inhibitory activity of both drugs (2.5 and 2.6 % of the isolates were resistant to AmB and L-AmB, respectively) and a close correlation between the MIC values of AmB and those of L-AmB (*R*^2^=0.61) with 98.2 % of all MICs for the two agents within ±2-fold dilutions of one another [14]. Here, we report the results of the first direct comparison of the susceptibility of L-AmB, tested using the CLSI BMD method, and AmB, tested using the Etest, against clinical isolates of *Candida* spp. Comparisons between two susceptibility testing results were undertaken in order to understand the utility of AmB Etest as a surrogate marker of L-AmB for making clinical decisions.

## Methods

### Clinical isolates

Between January 2000 and December 2013, a total of 598 clinical isolates of *Candida* spp. were collected from the bloodstream and other sterile sites of critically ill patients and those with haematological diseases. Each isolate represented a unique strain from a single patient, and was stored in glycerol at −80 °C until analysis. Prior to antifungal testing, they were sub-cultured on Sabouraud dextrose agar plates (bioMèrieux) to ensure the purity and viability of the cultures.

The species distribution was as follows: 248 *Candida albicans*, 190 *Candida parapsilosis sensu stricto*, 44 *Candida tropicalis*, 37 *Candida glabrata sensu stricto*, 33 *Candida orthopsilosis*, 15 *Candida guilliermondii*, 11 *Candida krusei*, 8 *Candida lusitaniae*, 6 *Candida norvegensis*, 2 each of *Candida dubliniensis* and *Candida kefyr* and 1 each of *Candida intermedia* and *Candida pelliculosa*. All the isolates were identified using standard procedures (morphology on cornmeal agar plates, germ-tube production in serum) and biochemical analyses [ID32C and VITEK-2 System (bioMérieux)]. *C. dubliniensis* was distinguished from *C. albicans* by the inability of the former to assimilate xylose and to grow at 42 °C. Identification of the *C. parapsilosis* and *C. glabrata* groups was achieved by molecular methods [15–17].

For this study, we did not use any additional data or samples other than those obtained through routine laboratory collection. Therefore, neither ethical approval nor patient consent was considered necessary. The data were analysed anonymously and were managed in accordance with the Italian data protection laws (privacy law).

### Antifungal susceptibility testing

L-AmB (Gilead Sciences) was obtained as a standard powder. BMD testing was performed in accordance with the CLSI method M27-A3 [18], using RPMI 1640 medium (Sigma), an inoculum concentration of 1.5×10^3^±1.0×10^3^ cells ml^−1^ and incubation at 35 °C. The final concentration of L-AmB ranged from 0.03 to 16 µg ml^−1^. MICs were determined visually in cultures incubated for 48 h [14], and were defined as the lowest concentration that inhibited 100 % of fungal growth compared with the drug-free controls.

The AmB Etest (AB BIODISK) was performed using RPMI-1640 agar plates (Biolife), as recommended in the manufacturer’s guidelines, with inoculum suspensions prepared in the same way as for the CLSI method. The Etest MICs were read at 24 h, or after 48 h if insufficient growth was present after 24 h, and were defined as the lowest drug concentration at which the border of the elliptical inhibition zone intercepted the scale on the antifungal strip. All tests were performed in duplicate and, in case of discrepancies, were repeated once more.

Each assay was validated using the quality control isolates *C. krusei* ATCC 6258 and *C. parapsilosis* ATCC 22 019 listed in CLSI [18].

### Analysis of the results

CLSI has not determined breakpoints for AmB [19]; in order to perform a comparison in this study, the isolates inhibited by L-AmB or AmB at ≤1 µg ml^−1^ were considered susceptible, as detailed in previous studies [11, 20]. ‘Resistant’ isolates were defined as isolates with MIC >1 µg ml^−1^. MIC data are presented as the range, MIC_50_ (MIC causing 50 % growth inhibition of the isolate), MIC_90_ (MIC causing 90 % growth inhibition of the isolate) and geometric mean (GM) of each species. MIC_50_ and MIC_90_ values were calculated for those species with 10 or more isolates. Concordance between the BMD and Etest results was expressed as either the essential agreement (EA) or the categorical agreement (CA). MIC discrepancies corresponding to no more than ±2-fold dilutions were used to calculate the EA. To this end, the Etest MIC values were rounded up to the next highest CLSI concentration corresponding to the twofold dilution series used in the BMD method. The CA was defined as the percentage of isolates classified in the same category by the two methods. Discrepant results were considered a ‘very major error’ whenever the strain was classified as susceptible by the Etest and resistant by the BMD (false susceptibility); a classification of resistance by Etest with a corresponding susceptible BMD pattern was considered a ‘major error’ (false resistance). The Mann–Whitney *U* test was applied to evaluate the significance of the differences in antifungal susceptibility determined by the two tests. Moreover, the correlation between the methods was assessed using Spearman’s rank correlation coefficient (ρ), and the respective *P* value plotting the MICs of AmB determined by the Etest *versus* those of L-AmB determined by the BMD method. The level of significance was defined as a *P* value less than 0.05. Statistical analyses were performed with GraphPad Prism version 5.0 for Windows.

## Results and discussion

The MICs for the quality control isolates were within the recommended limits [18, 19]. The AmB Etest reading ranges (µg ml^−1^) for the reference strains *C. krusei* ATCC 6258 and *C. parapsilosis* ATCC 22019 were 0.25 to 1 and 0.032 to 0.75, respectively. The MIC range (µg ml^−1^) of L-AmB for the same strains was 0.125 to 1 and 0.06 to 0.5, respectively.

Table 1 lists the MICs for the L-AmB BMD method and the AmB Etest, together with the EA and CA values obtained for each one when tested against each *Candida* spp. Table 2 shows the correlation between the results of the antifungal tests.

**Table 1.** *In vitro* susceptibilities of 598 clinical isolates of *Candida* spp. causing invasive candidiasis

Species (no. of isolates)	L-AmB BMD MIC (µg ml^−1^)		AmB Etest MIC (µg ml^−1^)		EA (%)	CA (%)	Categorical errors, no.
	MIC_50/90_	GM	MIC_50/90_	GM			
*C. albicans* (248)	0.25/0.5	0.26	0.19/0.38	0.21	96.4	100	–
*C. parapsilosis* *sensu stricto* (190)	0.25/1	0.30	0.125/0.5	0.18	88.5	97.9	3 VME – 1 ME
*C. tropicalis* (44)	0.5/2	0.61	0.38/0.75	0.41	97.7	90.9	4 VME
*C. glabrata* *sensu stricto* (37)	0.5/1	0.56	0.5/0.75	0.39	100	94.6	2 VME
*C. orthopsilosis* (33)	0.125/0.25	0.14	0.125/0.25	0.10	87.9	100	–
*C. guilliermondii* (15)	0.25/1	0.38	0.19/0.25	0.18	73.3	93.3	1 VME
*C. krusei* (11)	1/2	1	0.5/3	0.73	100	72.7	3 VME – 2 ME
*C. lusitaniae* (8)	na	0.65	na	0.12	37.5	87.5	1 VME
*C. norvegensis* (6)	na	0.32	na	0.16	66.6	83.3	1 VME
Other species* (6)	na	0.45	na	0.24	100	100	–

VME, very major error; ME, major error; na, not applicable.

*C. dubliniensis and *C. kefyr* (two isolates each); *C. intermedia* and *C. pelliculosa* (one isolate each).

**Table 2.** Plot of MICs between AmB, according to the Etest, and L-AmB, measured using the CLSI BMD method

AmB Etest MIC (µg ml^−1^)	No. of isolates with L-AmB BMD MIC (µg ml^−1^) of:							Total
	0.03	0.06	0.125	0.25	0.5	1	≥2	
≥2						3	2	5
1			2		4	8	5	19
0.75					5	17	3	25
0.5		1	1	6	22	16		46
0.38		1	4	17	23	5	1	51
0.25		3	11	58	26	7	2	107
0.19		2	34	52	40	7		135
0.125		3	44	51	19	6	2	125
0.094		4	17	19	8	2		50
0.064		1	8	3	1	1		14
0.047			2	4	2			8
0.032			6	3				9
0.016					1			1
0.004		1	1	1				3
Total		16	130	214	151	72	15	598

Spearman’s ρ=0.483; *P*<0.0001.

Both L-AmB activity measured by the BMD method and AmB activity measured by the Etest were similar to previously published values [10, 21, 22]. The 598 *Candida* strains generated L-AmB MICs (BMD method) that were within a span of six twofold dilutions (ranging from 0.06 to 2 µg ml^−1^), whereas a broad distribution of MICs was obtained using the same strains and AmB (Etest method), in which the values ranged from 0.004 to 3 µg ml^−1^. The differences in the MICs obtained by the two methods were significant (*P*<0.0001; Mann–Whitney *U* test) because the AmB MICs identified using the Etest were lower than the L-AmB MICs obtained using the BMD method (GM MICs of 0.20 and 0.31, respectively), but the test results were significantly correlated (ρ=0.48; *P*<0.0001).

The overall EA was 91.5 %, ranging from 37.5 % (*C. lusitaniae*) to 100 % (*C. glabrata* and *C. krusei*), and was similar to the values reported in other studies, even though the latter compared AmB results obtained using the CLSI method and the Etest [21, 23]. The low percentage agreement for *C. lusitaniae* (GM of 0.12 µg ml^−1^ and 0.65 µg ml^−1^ using the L-AmB BMD and AmB Etest, respectively) may have been due to the relatively small strain number tested for this *Candida* spp.

The absolute CA between the test results was 97.3 %. Fourteen isolates that were categorized as ‘likely resistant’ by BMD were not detected by Etest. This finding is therefore in contrast to previous observations [24, 25] in which the Etest was shown to be more sensitive than microdilution tests in detecting resistant isolates. The discrepancy may be due to differences in drug formulations, but remains to be elucidated.

In summary, this study is the first to compare L-AmB and AmB MICs obtained using the BMD method and the Etest, respectively. It showed that the Etest is a feasible and trustworthy alternative to the CLSI method for estimating the *in vitro* susceptibility of *Candida* spp. to L-AmB. Thus, the Etest can serve as a surrogate marker to predict the sensibility and resistance of *Candida* to L-AmB, and thereby to support the decision to use antifungal treatment. This study has several limitations. First, the lack of CLSI species-specific breakpoints precludes a more standard comparison for the assessment of the CA. Second, none of our strains were characterized with respect to resistance mechanisms. Third, the CLSI document M27 A-3 [18] does not include a guideline for testing the susceptibility of *Candida* to L-AmB, which made it difficult to compare the Etest and the reference method. However, our results provide a useful starting point for future comparisons.
